# Tratamentul cu laser YAG a tumorilor uroteliale de cale urinară superioară

**Published:** 2014

**Authors:** Niță Gh, Georgescu D, Mulțescu R, Draguțescu M, Mihai B, Persu C, Geavlete B, Geavlete P

**Affiliations:** * Spitalul Clinic de Urgență “Sf. Ioan”, București, România

**Rezumat**

**Introducere**. Tratamentul standard al carcinoamelor uroteliale de cale urinară superioară (UUT-UCCs) este reprezentat de nefroureterectomia totală cu cistectomie perimeatică. Scopul acestui studiu a fost analiza retrospectivă a factorilor care pot influența prognosticul pacienților UUT-UCCs tratați endoscopic. 

**Material și metodă**. In perioada 1998 - 2010 în cadrul Clinicii de Urologie a Spitalului Clinic de Urgență “Sf. Ioan” București au fost diagnosticați și tratați pentru UUT-UCCs un număr de 187 pacienți. Dintre aceștia la 65 cazuri s-a utilizat abordul endoscopic. Indicațiile pentru tratament endoscopic au fost imperative sau elective. Abordul retrograd (rigid sau flexibil) a fost utilizat la 47 cazuri, iar cel anterograd la 18 cazuri. Ablația tumorală s-a realizat utilizând electrorezecția sau un laser YAG. Protocolul de urmărire a inclus tomografie computerizată sau urografie intravenoasă, citologie urinară (cazuri selecținate), cistoscopie și ureteroscopie. S-a analizat retrospectiv rata de recidivă fiind identificați factorii care o influențează.

**Rezultate**. În timpul perioadei de urmărire un număr de 31 (47.6%) pacienți au dezvoltat recidive tumorale la nivelul tractului urinar sperior. La 20 cazuri (30.7%) s-au înregistrat recidive vezicale. Timpul median din momentul diagnosticului și până la apariția primei recidive locale a fost de 12.6 luni. 18 pacienți (27.69%) au necesitat ulterior de nefroureterectomie. Ratele de supraviețuire fără recidivă la 1, 3 și respectiv 5 ani de la diagnostic au fost de 61% (40 pacienți) , 55.3% (36 pacienți) respectiv 52.3% (34 pacienți). Rata de recidivă pentru tumorile pielo-caliceale a fost de 53.84% (21 din 39 cazuri) și doar 45.45% (10 din 26 cazuri) pentru localizările ureterale. Rata de recurență pentru tumorile low-grade a fost de 36,36% (16 din 44 cazuri) și de 71.42% (15 din 21 cazuri) pentru tumorile high-grade. Tumorile peste 1.5 cm s-au însoțit de o rată a recurențelor semnificativ mai ridicată (mai ales recurențe locale) comparativ cu cele sub 1.5 cm (64.2 versus 43.13%).

**Concluzii**. Cei mai importanți factori de prognostic pentru evoluția UUT-UCCs sunt reprezentați de localizare, dimensiune și mai ales gradingul tumoral. Complianța pacienților este foarte importantă pentru depistarea recidivelor.

**Cuvinte-cheie:** laser YAG, tumoare urotelială, carcinoame uroteliale

